# A real‐world study on the prevalence and risk factors of medication related osteonecrosis of the jaw in cancer patients with bone metastases treated with Denosumab

**DOI:** 10.1002/cam4.6429

**Published:** 2023-08-09

**Authors:** Paola Bracchi, Ernesto Zecca, Cinzia Brunelli, Rosalba Miceli, Gabriele Tinè, Massimo Maniezzo, Silvia Lo Dico, Mariangela Caputo, Morena Shkodra, Augusto T. Caraceni

**Affiliations:** ^1^ Palliative Care, Pain Therapy and Rehabilitation Unit Fondazione IRCCS Istituto Nazionale dei Tumori Milano Italy; ^2^ Biostatistics for Clinical Research Fondazione IRCCS Istituto Nazionale dei Tumori Milano Italy; ^3^ Odontostomatology Unit Fondazione IRCCS Istituto Nazionale dei Tumori Milano Italy; ^4^ Institute of Clinical Medicine University of Oslo Oslo Norway; ^5^ Department of Clinical Sciences and Community Health Università degli Studi di Milano Milan Italy

**Keywords:** bone metastasis, cancer, Denosumab, osteonecrosis of the jaw, risk factors

## Abstract

**Aim:**

Assessing the incidence of Medication Related Osteonecrosis of the Jaw (MRONJ) in cancer patients with bone metastases receiving Denosumab (Dmab) and identifying potential risk factors.

**Methods:**

A retrospective observational study on consecutive cancer patients with bone metastases, who received at least one dose of Dmab and one follow‐up visit. MRONJ crude cumulative incidence (CCI) was estimated considering death without MRONJ as competing event. Multiple regression models were used to study the association between MRONJ incidence and potential risk factors: age, cancer diagnosis, previous bisphosphonates, dental treatments before starting Dmab, extraction or other dental treatment during Dmab, chemotherapy, hormone therapy, and antiangiogenic (AA) agents concurrent use.

**Results:**

On 780 patients included (median follow‐up 17 months), 54% and 18% had, respectively, breast and prostate cancer. The mean number of Dmab administration was 12. Fifty‐six patients developed MRONJ with a 24‐ and a 48‐month crude cumulative incidence of 5.7% (95% Cl: 4.2%–7.8%) and 9.8% (95% CI: 7.6%–12.7%), respectively. Higher MRONJ incidence was significantly associated with middle aged group (>56 and ≤73), both at univariate and multivariate analysis (*p* = 0.029 and 0.0106). Dental treatments (Hazard Ratio [HR] = 3.67; *p* = 0.0001), dental extractions (HR = 23.40; *p* < 0.0001), and previous BP administration (HR = 2.62; *p* = 0.0024) were significantly associated with higher MRONJ incidence at multivariate Cox analysis. Although not statistically significant, MRONJ incidence was lower for patients receiving chemotherapy or hormone therapy and higher for those receiving AAs.

**Conclusions:**

The results confirm a clinically relevant incidence of Dmab‐induced MRONJ. Dental treatments, especially extraction, during and before Dmab, constitute a serious risk factor. The role of AA concurrent administration deserves further investigations.

## 1 INTRODUCTION

Bone is one of the main sites of metastasis in patients with advanced cancer, with an incidence as high as 60%–75% in malignancies like breast and prostate cancer.^1^ The presence of bone metastases can be complicated by the onset of skeletal‐related events (SRE).^2^ Bone‐modifying agents (BMAs) are commonly used for preventing and managing such events. The main BMAs used for osteoclast activity inhibition and bone health maintenance in patients with bone metastasis are bisphosphonates (BP) and denosumab (Dmab), both proven to be effective in reducing SREs.^3^, ^4^, ^5^ However, BMAs also have side effects, including medication related osteonecrosis of the jaw (MRONJ)^6^, ^7^ a drug‐limiting side effect which causes an important impairment to a patient's overall performance. In patients receiving BP treatment, MRONJ is estimated to have an incidence of <0.5% according to a 2017 Cochrane Review^8^ but, a recent review reported MRONJ frequencies in metastatic bone and myeloma patients ranging from 1% to 15% or more.^9^

Dmab is a human monoclonal antibody directed against the receptor activator of nuclear factor‐κβ ligand (RANKL), which has become widely used in cancer patients only in the last decade, and therefore, its side effects including MRONJ incidence are not yet completely appreciated. Evidences indicate that Dmab could perform better than BP for SRE prevention,^10^ with no renal toxicity and less infusion related toxicity. However, a slightly higher incidence of MRONJ has been reported for Dmab.^4^, ^5^, ^8^, ^11^ Currently, evidence from clinical practice^12^, ^13^, ^14^, ^15^, ^16^, ^17^, ^18^, ^19^, ^20^, ^21^, ^22^, ^23^, ^24^, ^25^ in oncological settings is based mainly on small patient samples^14^, ^15^, ^16^, ^17^ reporting on bone‐modifying agents in general, without clarifying Dmab and eventual risk factors role in the development of MRONJ.^18^, ^19^, ^20^, ^21^, ^22^, ^23^, ^24^, ^25^, ^26^ Only few real‐world studies^13^, ^14^, ^15^, ^16^, ^17^ focused on MRONJ related to Dmab administration.

The aim of the present study is to assess the incidence of MRONJ in oncological patients with bone metastases receiving Dmab and to evaluate the role of potential, concomitant local or systemic risk factors.

## 2 METHODS

### 2.1 Study design and population

This is a retrospective observational study carried out on consecutive cancer patients with bone metastases attending the Palliative Care and Pain Outpatient Clinic at a tertiary oncological center from April 2013 to September 2018. Patients who had received at least one dose of Dmab and had at least one follow‐up visit were eligible.

### 2.2 Denosumab administrations (regimen)

All patients received 120 mg of subcutaneous Dmab every 28 days. Patients were scheduled to receive at least 24 administrations but this could be reduced or extended depending on disease characteristics and occurring side effects.

Before starting Dmab, all patients underwent preventive dental care, including:

- examination by a dental specialist, with expertise in the clinical management of MRONJ, to exclude the need for dental treatments or the presence of risk factors for MRONJ. A panoramic dental x‐ray was also requested before the examination. When needed, a preventive dental treatment (ie. dental extraction/s) was performed before starting Dmab. In this case, Dmab administration was delayed for at least 4–6 weeks after the intervention and depending on the type of intervention, prophylactic antibiotic therapy was prescribed,
- regular maintenance oral hygiene recommendations.

Blood levels of calcium and vitamin D were also tested, and in case of abnormal values, supplementary therapies were administered before starting Dmab.

During Dmab treatment, blood calcium and vitamin D levels were regularly checked every 4 weeks and clinical dental examination together with an panoramic dental x‐ray (when required by the dentist), were performed every six administrations.

### 2.3 Diagnosis of MRONJ

MRONJ clinical diagnosis was based on the guidelines of the American Association of Oral and Maxillofacial Surgeons.^27^ The diagnosis was made at routine scheduled dental examinations, or by referral in case of clinical suspicion, based also on panoramic dental x‐ray or CT scan when necessary. MRONJ diagnosis was ultimately confirmed by a dental specialist. Even if not fully answering to the AAOMS definition, we considered the cases classified as “stage 0” by AAOMS (symptomatic but without bone exposure or fistula) as MRONJ cases, considering also the evidence of progression to other stages.^27^, ^28^

### 2.4 Data collection

The list of patients who had received at least one dose of Dmab during the study period was obtained from the hospital administrative database. The following baseline data were retrieved from the electronic medical records: age, sex, primary tumor diagnosis, previous use of BP and, in this case, type and date of last BP administration, previous history of MRONJ, dental treatments before starting Dmab, presence of diabetes and previous administration of antiangiogenic agents (AA). Data related to the Dmab administration period included concurrent chemotherapy and hormone therapy, corticosteroids therapy for at least three consecutive months, anemia (defined as Hb values lower than 10 g/dL for at least three consecutive months), dental treatment received and type, as well as number of Dmab administrations.

For patients who developed MRONJ, the following data were recorded: MRONJ site, MRONJ staging, MRONJ treatment (ozone therapy and/or surgical intervention), outcome (healed/not healed) and whether treatment with Dmab or BP was resumed.

The study was approved by the institutional research Ethics Committee (identification number INT 191/18). Due to the retrospective nature of the study, informed consent was not requested.

### 2.5 Statistical analysis

Analyses of association between MRONJ occurrence and baseline variables were performed by estimating MRONJ crude cumulative incidence (CCI) curves. Time was computed from the date of Dmab start and MRONJ or death in the absence of MRONJ (competing event), whichever occurred first. In the absence of MRONJ or death, time was censored at the date of the last follow‐up. Univariate comparison between CCI curves according to categories of (time independent) baseline variables was performed using the Gray test.^29^ Cox models were applied to perform multivariable analyses and univariable analyses of time‐dependent variables, which are those changing over time during the follow‐up period. For the time‐dependent variables dental extraction, dental treatments, and AA administration, the only change considered was at the date of the first procedure.

One important variable to be taken into account in the multivariate Cox model was the number of DS doses, that is a time‐dependent variable continuously changing its values with time, differently from the other above mentioned time‐dependent variables (as for instance dental extraction), that only change the value once. Since investigating its role was not the main aim of our analysis, the DS dose number was included in the multivariate Cox model as fixed (not time‐dependent) baseline variable, that can be methodologically acceptable if considering it as an adjustment factor.

The intrinsic nature of time‐dependent variables hampered estimation of CCI curves which would treat them as baseline variables. In the Cox models, deaths in the absence of MRONJ were considered censored observations; this allowed to estimate the MRONJ‐specific hazard ratios (HR) and corresponding 95% confidence intervals (95% CI). Patient's age was modeled as continuous variables using three‐knot restricted cubic splines.

The analyses were carried out using R software (https://cran.r‐project.org, last access 19 July, 2023, R version 4.2.2 Copyright (C) 2022 The R Foundation for Statistical Computing).

## 3 RESULTS

### 3.1 Sample characteristics

A total of 814 consecutive patients with bone metastases received at least one dose of Dmab during the study period; 34 of them (4%) were lost to follow‐up after one single dose and were not included in this analysis. Baseline demographic and clinical characteristics of the 780 patients included are described in Table 1. The average age was 65 years (range 22–91), and the most common diagnosis were breast (54%) and prostate cancer (18.%). All patients had stage IV disease and multiple or single bone metastases.

**TABLE 1:** Baseline sociodemographic and clinical characteristics of the study participants (*N* = 780).

Sex	
Female *n* (%)	495 (63.5)
Male *n* (%)	285 (36.5)
Age	
Median (1st–3rd quartile)	65.0 (56.0–63.9)
Primary tumor	
Breast *n* (%)	422 (54.1)
Genito‐urinary *n* (%)	40 (5.1)
Lung *n* (%)	76 (9.8)
Melanoma *n* (%)	18 (2.3)
Prostate *n* (%)	143 (18.3)
Other *n* (%)	81 (10.4)
Diabetes	
Yes *n* (%)	55 (7.1)
No *n* (%)	725 (92.9)
Previous antiangiogenic (AA) therapy(*)	
Yes *n* (%)	35 (4.5)
No *n* (%)	745 (95.5)
Preventive dental treatments	
Yes *n* (%)	71 (9.1)
No *n* (%)	709 (90.9)
Previous bisphosphonate	
Yes *n* (%)	148 (19.0)
No *n* (%)	632 (81.0)
Previous MRONJ	
Yes *n* (%)	4 (0.05)
No *n* (%)	776 (99.5)

*Note*: (*) started before Dmab.

Nineteen percent of the patients (148) had received prior therapy with oral or intravenous BP: 96% zoledronic acid, 4% intravenous or oral ibandronate. The median time (interquartile range ‐IQR) between BP last and Dmab first administration was 2.8 (1.1–14.6) months, with 26 patients starting <1 month after. Four patients had previously developed MRONJ, already resolved before starting Dmab, none of them developed MRONJ after Dmab. Seventy‐one patients out of the 780 analyzed (9%) had undergone preventive dental treatments such as oral hygiene, root canal treatment or extraction, before starting Dmab.

The median number of Dmab administrations was 12 (IQR: 6–24). The median treatment duration was 12.4 (IQR: 5.1–23.7) months.

Table 2 describes disease and treatment related characteristics during DMAB treatment. A total of 121 patients (15.5%) developed anemia and 217 (27.4%) had been receiving corticosteroids for at least three consecutive months as continuous therapy, mainly for the management of symptoms such as pain, fatigue, and anorexia. Hormone therapy was given concurrently in 426 patients (54.6%), chemotherapy in 351 patients (45%) while 50 patients (6.4%) received antiangiogenic drugs: 35 of them were already on treatment prior to starting Dmab and continued it, while 15 started treatment during Dmab. Sunitinib was the most commonly used targeted agent in 14 cases, followed by sorafenib (10 cases). Other AAs used included cabozantinib, axitinib, pazopanib, regorafenib, and afatinib. The sum is higher than 100% because during the period of the follow‐up, any of the above associated treatments could have been interrupted and substituted with another one, according to the disease status and oncological treatment guidelines. However, these were not concomitant, but subsequent treatments during time. During Dmab, 53 patients (6.8%) underwent tooth extraction and 138 patients (17.7%) underwent other dental treatment (i.e., professional dental hygiene, fillings, and root canal treatment). Dental treatments and tooth extractions were performed under antibiotic therapy when necessary.

**TABLE 2:** Clinical characteristics during Dmab administration.

Number of doses	
Median (IQR)	12.0 (6.0–24.0)
Mean (min‐max)	14.5 (1–48)
Duration of Denosumab in months	
Median (IQR)	12.4 (5.1–23.7)
Mean (min‐max)	10.25 (1–125)
Dental extraction	
Yes *n* (%)	53 (6.8)
No *n* (%)	727 (93.2)
Dental treatment	
Yes *n* (%)	138 (17.7)
No *n* (%)	642 (82.3)
Antiangiogenic (AA) therapy (**)	
Yes *n* (%)	15 (1.9)
No *n* (%)	765 (98.1)
Hormone therapy	
Yes *n* (%)	426 (54.6)
No *n* (%)	354 (45.4)
Chemotherapy	
Yes *n* (%)	351 (45.0)
No *n* (%)	429 (55.0)
Corticosteroids	
Yes *n* (%)	214 (27.4)
No *n* (%)	566 (72.6)
Anemia	
Yes *n* (%)	121 (15.5)
No *n* (%)	659 (84.5)

*Note*: (**) started during Dmab treatment.

Abbreviation: IQR, interquartile range.

### 3.2 MRONJ incidence and associated factors

**FIGURE 1:**
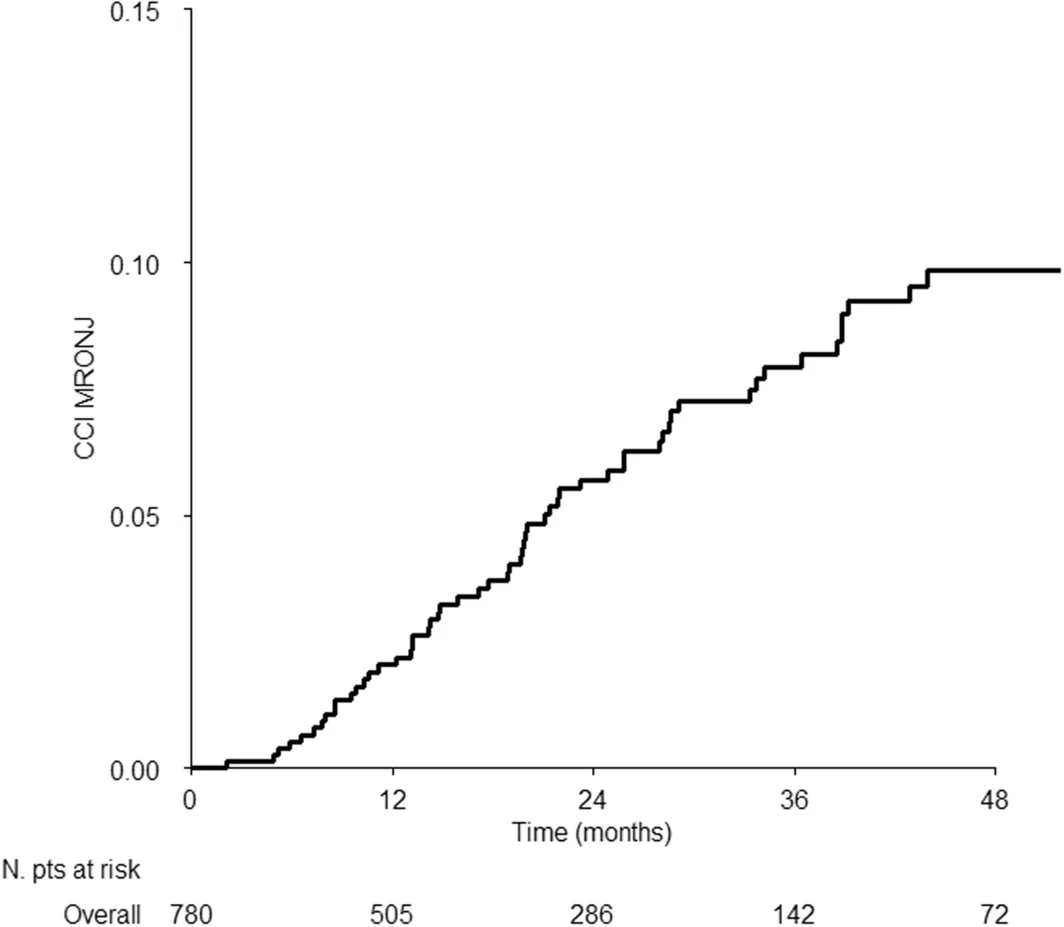
Cumulative incidence curve of medication related osteonecrosis of the jaw in the whole series.

Median follow‐up was 37.3 (IQR: 21.4–53.9) months, and was slightly different in patients without and with prior BP (34.6 [20.0–51.4] months and 52.4 (35.7–60.4) months, respectively). Fifty‐six patients developed MRONJ out of 780 (7.15%) with a 24‐, 36‐, and a 48‐month crude cumulative incidence of 5.7% (95% CI 4.2%–7.8%), 7.9% (95% CI 6.0%–10.4%), and 9.8% (95% CI 7.6%–12.7%), respectively (Figure 1).

Three patients resumed Dmab after MRONJ occurrence. 427 patients died for all causes, 402 of which without developing MRONJ.

**FIGURE 2:**
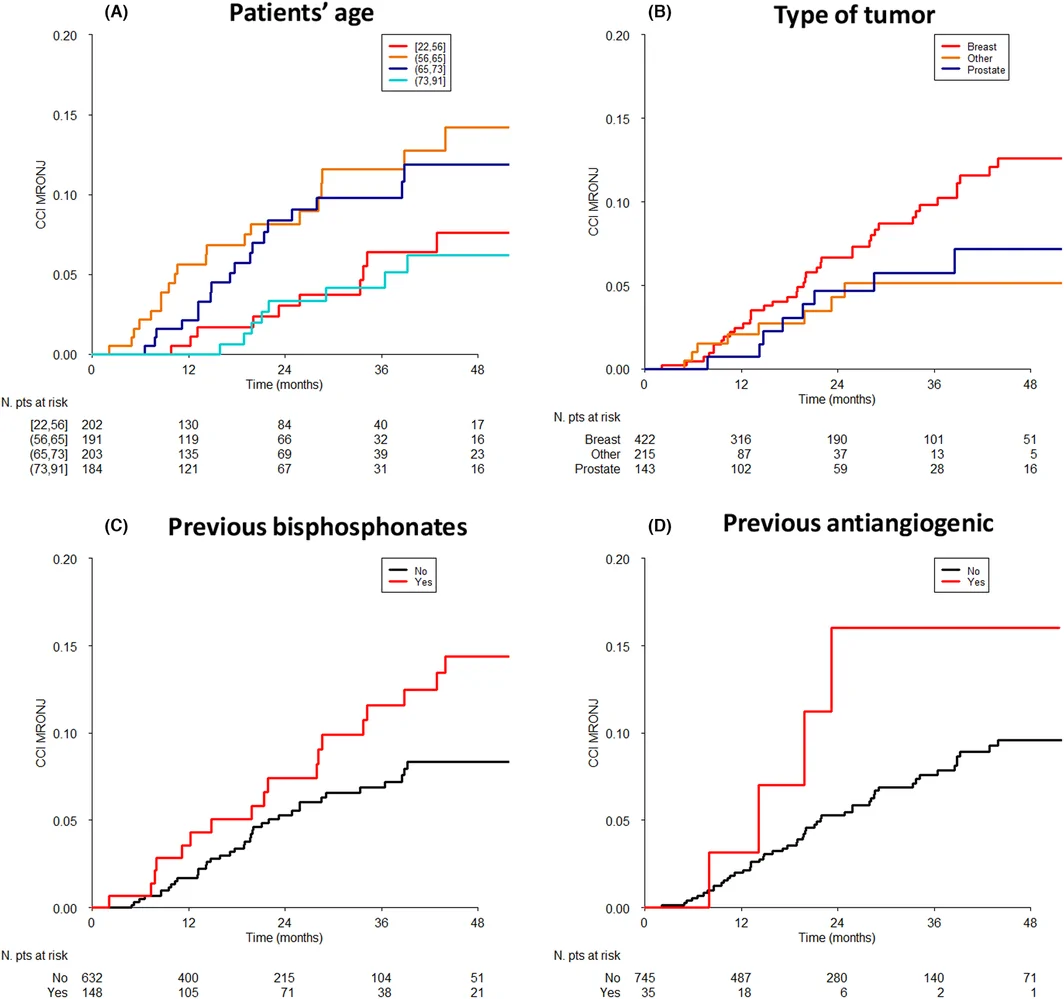
Crude cumulative incidence curves of MRONJ by selected characteristics. (A) patients' age (*p* value at Gray test = 0.0288); (B) primary tumor (*p* = 0.0972); (C) previous bisphosphonates (*p* = 0.0646); (D) previous antiangiogenetics (*p* = 0.1659).

The univariable association of different baseline patients and disease characteristics and treatments with the MRONJ incidence is shown in Figure S1 and in Figure 2; the former reports the 24‐ and 48‐month MRONJ incidence estimates, while the latter shows the whole incidence curves only for selected characteristics. MRONJ occurrence was significantly associated with patients' age (*p* = 0.029); younger (≤56 years old) and older (>73) patients presented with similar MRONJ incidence, which was lower as compared with the middle age class (>56 and ≤73) (Figure 2A). None of the additional variables achieved statistical significance (Figure S1).

Despite a non‐significant test result, good separation between the incidence curves, suggesting a potential clinical association with MRONJ incidence, was shown for tumor site (breast cancer patients showed the highest MRONJ incidence as compared with other tumor sites; *p* = 0.0972; Figure 2B), previous BP administration (*p* = 0.0636; Figure 2C), and previous AA drugs (*p* = 0.1659; Figure 2D). As regard to time‐dependent variables, univariable Cox analysis are displayed in the first part of Table 3. Dental treatments (HR = 4.18; 95% CI: 2.40–7.25) and dental extraction (HR = 30.60; 95% CI: 17.66–53.03) were significantly associated with MRONJ occurrence (*p* < 0.0001 for both). Concomitant or follow‐up AA drug assumption was not significant (*p* = 0.1369) but, noteworthy, it was associated with doubled MRONJ risk.

**TABLE 3:** Results of the univariate Cox models analyzing the association between MRONJ and time‐dependent variables and results of the multivariate Cox models analyzing the association between MRONJ and selected variables.

	Hazard ratio (95% confidence interval)	*p* value at Wald Test
Univariate Cox models		
Antiogenetic treatment yes (*) versus no	2.01 (0.80,5.04)	0.1369
Dental extraction yes versus no	30.60 (17.66,53.03)	<0.0001
Other dental treatments yes versus no	4.18 (2.40, 7.25)	<0.0001
Multivariate Cox model		
Age (years)*		0.0106
56 versus 30	3.22 (0.70, 14.82)	
65 versus 30	3.01 (0.52, 17.56)	
73 versus 30	1.58 (0.30, 8.45)	
Previous Bisphosphonate Yes versus no	2.62 (1.40, 4.88)	0.0024
Type of Tumor		0.5024
Prostate versus breast	0.83 (0.37, 1.89)	
Other versus breast	0.56 (0.21, 1.53)	
Dental treatments before starting Dmab Yes versus no	1.26 (0.59, 2.68)	0.5517
Antiangiogenetic treatment yes (***) versus no	3.11 (0.94, 10.26)	0.0621
Dental extractions yes versus no	23.40 (12.98, 47.18)	<0.0001
Dental treatments Yes versus no	3.67 (1.95, 6.88)	0.0001
Number Dmab doses		<0.0001
6 versus 12	1.87 (1.15, 3.06)	
6 versus 24	9.11 (4.13, 20.09)	

*Note*: (*) Concomitant or follow‐up initiated antiangiogenetic drug assumption. (**) 56, 65, and 73 are, respectively, the first quartile, the median and the third quartiles of the age distribution. (***)Concomitant or follow‐up initiated antiangiogenetic drug assumption.

Cox multivariable analysis (Table 3, second part) confirms the univariable results for time‐dependent variables and for age (Figure 2 and Figure S1) and demonstrates a significant association for previous BP administration (HR = 2.62; 95% CI: 1.40–4.88; *p* = 0.0024). In the same Cox multivariate model, the association between MRONJ incidence and the number of Dmab doses was statistically significant with lower doses associated with higher MRONJ risk (HR 6 vs. 24 doses = 9.11; 95% CI: 4.13–20.09; *p* < 0.0001).

#### 3.2.1 Description of patients developing MRONJ

Among 56 patients developing MRONJ, the mandible was the most common site (64%), followed by the maxilla (32%) and both in only 2 cases (4%). In three cases (6%), a stage 0 MRONJ was diagnosed, whereas 24 patients (43%) had stage 1 of MRONJ, 26 (45%) stage 2, 2 (4%) stage 3 MRONJ, and in one patient the staging of MRONJ was not available. As specified in the methods section, the AAOMS staging system was used.^27^

After MRONJ diagnosis, four patients were lost to follow‐up. The remaining 52 patients, 40 (77%) underwent ozone therapy, 27 (52%) surgery with sequestrectomy of the necrotic area and three patients were still awaiting treatment (Table S1).

At the end of the follow‐up period, 23 patients were still undergoing MRONJ treatment or lost to follow up, while 29 had completed MRONJ treatment and reported full MRONJ resolution. After MRONJ resolution, nine of these patients resumed Dmab (five patients) or BPs (four patients). The decision to resume BMAs was made based on the disease characteristics, symptoms presented, and patient's preferences. Specifically, in seven patients with progressive bone disease and two with hypercalcemia, treatment was proposed and resumed with their consent. In 20 cases, treatment was withdrawn, in seven of them because of worsening clinical conditions associated with disease progression, in other seven who had stable bone disease treatment was not proposed, while six patients with indication to resume BMAs refused.

## 4 DISCUSSION

In this study, we have investigated the incidence of MRONJ in patients receiving Dmab for bone metastases and described factors associated with a higher incidence. Out of 780 patients, 56 developed MRONJ, with a 24‐ and 48‐month crude cumulative incidence of 5.7% (95% CI: 4.2%–7.8%) and 9.8% (95% CI: 7.6%–12.7%), respectively.

Literature data on MRONJ incidence are variable.^13^, ^14^, ^15^, ^16^, ^17^ A single‐center retrospective study performed in France on 141 patients reported MRONJ incidence of 3% at 12 months of treatment and 7% and 8%, respectively, at 24 and 30 months.^13^ Other studies on real‐world data have reported incidences as high as 12.6%^15^ and 13.6%.^16^ In phase 3 trials, incidences were as low as 0.7%–1.9%,^3^, ^5^ but significantly higher in the open label extension of Stopeck trial.^5^, ^30^ A recent systematic review and meta‐analysis of randomized controlled trials has reported an incidence of MRONJ in cancer patients under treatment with Dmab which ranged from 0.5% to 2.1% after 1 year, 1.1% to 3.0% after 2 years, and 1.3% to 3.2% after 3 years of exposure.^31^

Similarly to previous reports,^15^, ^27^, ^32^, ^33^ at multivariate analysis (Table 3), we found MRONJ to be significantly associated with dental treatments (HR = 3.67; 95% CI: 1.95, −6.88; *p* = 0.0001), especially dental extraction (HR = 23.40; 95% CI: 12.98–47.18; *p* < 0.0001) which was found to be the most common event preceding MRONJ onset. In fact, 28 (50%) out of the 56 MRONJ patients had previously had at least one extraction.

Patients age (*p* = 0.0106) and previous BP administration (*p* = 0.0024) were also identified as significantly associated factors. However, results on the significance of previous BP administration and MRONJ incidence from other studies are mixed, although there is some evidence suggesting a higher prevalence of MRONJ for sequential BP‐Dmab therapy.^17^, ^34^, ^35^ The number of Dmab doses was statistically significant with a trend toward a decreasing MRONJ risk at increasing dose levels, possibly explained by predominance of patients with higher risk at shorter follow‐up times. However, this result has to be interpreted with caution as the number of Dmab doses was modeled as fixed (not time‐dependent) variable to be adjusted for and it was not the main study objective. Other authors have reported development of MRONJ after an average of 14–15 doses of Dmab^36^, ^37^ but without showing any significant associations.

MRONJ has been described as a multifactorial disease with both systemic and local factors involved in its development. Among systemic factors, comorbidities such as diabetes and anemia and the use of concomitant medications like corticosteroid therapy, AAs and others,^38^ have been associated to the risk of developing MRONJ. In our study, we did not find any significant association between the abovementioned factors and MRONJ incidence. Yet, the lack of statistically significant association could also be due to the low number of patients with diabetes, anemia or receiving corticosteroids in our study. As to AA drugs, while their administration was not significantly associated with MRONJ, MRONJ cases were doubled in patients receiving this type of drug. Previous evidences^39^, ^40^ have suggested a role of AA agents in the occurrence of osteonecrosis, especially when administered together with antiresorptive drugs such as Dmab. This could be partially explained by their mechanism of action, in suppressing vascular regeneration that could facilitate MRONJ.^36^, ^41^ Altogether these findings indicate a potential role of AA agents in MRONJ risk, warranting further research.

MRONJ incidence among younger and older patients was lower than among intermediate age groups (56–65 years old) (Figure 2). Previous studies have reported association of MRONJ with patients age, but differently from our findings, MRONJ incidence increased with age.^15^, ^24^ This finding is difficult to explain in the absence of more detailed information on other potential individual risk factors (ex. presence of periodontitis).

We did not find any statistically significant sex differences, unlike a previous study that showed a correlation between MRONJ incidence and sex,^14^ with females being affected more than males.

To the best of our knowledge, this study is among the few estimating the incidence of MRONJ among cancer patients receiving Dmab in clinical practice, and despite the limitations due to its retrospective and single‐center nature, the sample size is considerable. One of the main results is the strong association between MRONJ onset and dental extraction or other dental intervention. The diagnosis of MRONJ was made by a dental specialist, experienced in the diagnosis and management of MRONJ, using the staging system of the AAOMS guidelines.^27^ We acknowledge the debate about the controversial AAOMS definition^42^ and the correlated risk of underestimation; very probably the few cases of “stage 0” (3 cases, 6%), included after evaluation by a MRONJ specialist and CT scan examination, do not change the results of our study. Additionally, patients at risk for or with established osteonecrosis can also present with other underlying factors and comorbidities exacerbating or contributing to the disease, which are not necessarily medication related; therefore, the interpretation of causal factors could be challenging.^42^ Due to retrospective nature of the study, information about the onset of diabetes and anemia as well as the administration interval for chemotherapy, hormonotherapy, and corticosteroids was lacking. For this reason, it was not possible to adjust models for the above variables. The lack of a control group and the unknown duration of previous BP therapy are both important limitations of the present study.

## 5 CONCLUSIONS

Incidence of MRONJ according to our data study is higher than that reported in the published guidelines.^27^ MRONJ incidence was higher in patients receiving dental treatments, especially dental extraction during Dmab therapy. The use of a previous BP and time span between the previous BP and the start of Dmab, as well as the need of preventive dental treatment before starting Dmab were also identified as potential risk factors. Accordingly, providing effective preventive dental care, close collaborative oral examination and regular maintenance of oral hygiene by oral specialists, as suggested by published experiences^43^, ^44^, ^45^ could help in reducing the need of dental treatments and possibly the risk of MRONJ onset.

Given the continuous spread of the application of new target therapies, it will be important to establish if and how they can contribute to MRONJ development. Prospective and larger sample studies are required for better understanding the role of local and systemic risk factors that favor MRONJ development.

## AUTHOR CONTRIBUTIONS

**Paola Bracchi:** Conceptualization (equal); data curation (lead); investigation (lead); methodology (supporting); writing – original draft (equal); writing – review and editing (equal). **Ernesto Zecca:** Conceptualization (equal); data curation (supporting); investigation (supporting); methodology (supporting); validation (lead); writing – original draft (equal); writing – review and editing (equal). **Cinzia Brunelli:** Conceptualization (equal); data curation (supporting); formal analysis (supporting); methodology (supporting); project administration (lead); visualization (supporting); writing – original draft (equal); writing – review and editing (equal). **Rosalba Miceli:** Conceptualization (supporting); data curation (supporting); formal analysis (lead); methodology (equal); visualization (lead); writing – review and editing (equal). **Gabriele Tinè:** Formal analysis (supporting); methodology (supporting); visualization (supporting); writing – review and editing (equal). **Massimo Maniezzo:** Conceptualization (equal); data curation (equal); investigation (supporting); validation (equal); writing – original draft (supporting). **Silvia Lo Dico:** Data curation (supporting); investigation (supporting); writing – review and editing (equal). **Mariangela Caputo:** Data curation (supporting); investigation (supporting); writing – review and editing (equal). **Morena Shkodra:** Conceptualization (supporting); formal analysis (supporting); writing – original draft (equal); writing – review and editing (equal). **Augusto T. Caraceni:** Conceptualization (equal); formal analysis (equal); project administration (supporting); resources (lead); supervision (lead); writing – original draft (lead); writing – review and editing (equal).

## FUNDING INFORMATION

This research did not receive any specific grant from funding agencies in the public, commercial, or not‐for‐profit sectors.

## CONFLICT OF INTEREST STATEMENT

AC has received honoraria from Angelini, Shionogi, Kyowa Kirin, Molteni, Pfizer/ Eli Lilly Italia Spa, Mundipharma. EZ has received honoraria from Amgen. All other authors declare that they have no known competing financial interests or personal relationships that could have appeared to influence the work reported in this paper.

## Supporting information


Figure S1.
Table S1.

## Data Availability

The data that support the findings of this study are available from the corresponding author upon reasonable request.
