# Supplementary material for: A real‐world study on the prevalence and risk factors of medication related osteonecrosis of the jaw in cancer patients with bone metastases treated with Denosumab
Source: Cancer Med. 2023 Aug 9;12(17):18317–26. doi: 10.1002/cam4.6429 (PMC10523953; doi:10.1002/cam4.6429)
Supplement: Supplementary file 1 — Figure S1. Table S1. [file CAM4-12-18317-s001.docx]

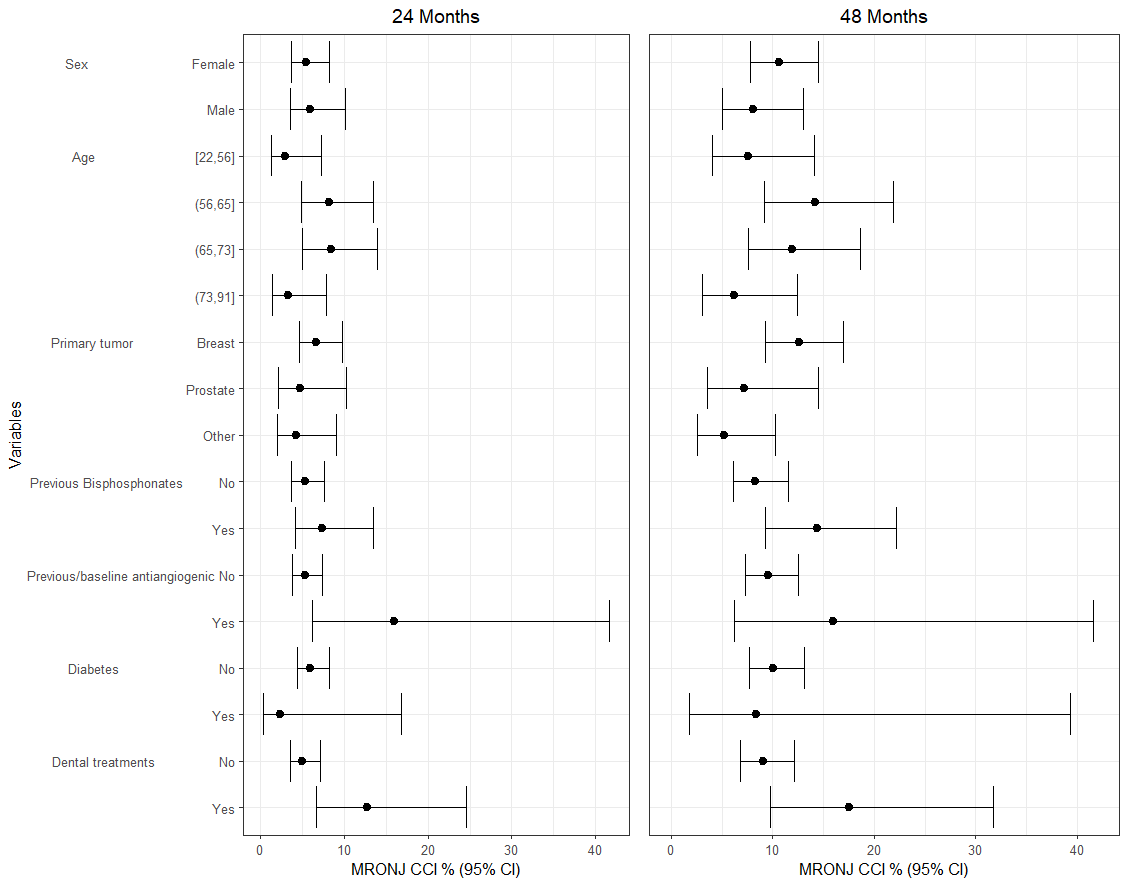


**SUPPLEMENTARY FIGURE 1.** *Crude cumulative incidence estimates of MRONJ at 24 and 48 months according to different baseline patients and disease characteristics and treatments.*

*Patients’sex p value at Gray test: =0.5667; patients’ age p=0.0288; primary tumor p=0.0972; previous bisphosphonates p=0.0636; previous antiangiogenetics p=0.1659; diabetes p=0.3716; dental treatments p=0.0139.*

**Supplementary Table 1 Results of MRONJ** treatment therapy (N=52)*

| **MRONJ** | | **Ozone Therapy** | **Surgery** | **Resolution** | |
| --- | --- | --- | --- | --- | --- |
| **Stage** | **No. of patients** |  |  | **Yes** | **Missing or still undergoing treatment** |
| 0  1  2  3 | 2  6  12  0 | Yes | Yes | 11 | 9 |
| 0  1  2  3 | 1  10  8  1 | Yes | No | 10 | 10 |
| 0  1  2  3 | 0  5  2  0 | No | Yes | 7 | 0 |
| 0  1  2  3  Unknown | 0  0  4  0  1 | No | No | 1 | 4 |

** 4 patients were lost to follow-up before starting any treatments for MRONJ.*
